# Case report: A case of oviductal and uterine leiomyosarcoma in an 11-year-old dog

**DOI:** 10.3389/fvets.2023.1227799

**Published:** 2023-12-07

**Authors:** Jillian Kazmierczak, Nicole J. Sugai, Katie E. Withowski, Abe Jonatan, Tanya LeRoith, Julie T. Cecere

**Affiliations:** ^1^DVM Candidate 2023 Virginia-Maryland College of Veterinary Medicine, Blacksburg, VA, United States; ^2^Department of Small Animal Clinical Sciences, Virginia-Maryland College of Veterinary Medicine, Blacksburg, VA, United States; ^3^Locum Department of Small Animal Clinical Sciences, Virginia-Maryland College of Veterinary Medicine, Blacksburg, VA, United States; ^4^Department of Biomedical Sciences and Pathobiology, Virginia-Maryland College of Veterinary Medicine, Blacksburg, VA, United States

**Keywords:** oviductal leiomyosarcoma, uterine leiomyosarcoma, uterus, oviduct, oviductal mass, uterine mass, neoplasia, dog

## Abstract

An 11-year-old, intact female Pomeranian dog was presented for evaluation due to an 18-h history of anorexia and lethargy. Abdominal ultrasound revealed a 3×3 cm mass of mixed echogenicity at the level of the left ovary. At laparotomy, a 5 mm mass was identified at the cranial region of the right uterine horn and a 3 cm round mass was visualized near the cranial aspect of the left uterine horn. Ovariohysterectomy was performed. A diagnosis of grade 1 oviductal and uterine leiomyosarcoma was made via histopathology for both masses. Oviductal leiomyosarcomas are rare and generally locally invasive similar to other soft tissue sarcomas but do not often metastasize. Uterine leiomyosarcomas are also uncommon but are one of the more common tumors affecting the female reproductive tract. This is the only known case report of oviductal leiomyosarcoma in the dog and the only report of uterine leiomyosarcoma in addition to oviductal leiomyosarcoma as well. This case illustrates the oviduct as an additional site that can be affected by leiomyosarcoma and demonstrates surgery as a treatment option for patients diagnosed with this condition.

## Background

Female reproductive tract neoplasms are an uncommon finding in small animal veterinary medicine. Ovarian tumors only have a prevalence of 0.5%–1.2% in the dog (1). Uterine tumors are even more uncommon making up only 0.3%–0.4% of all canine and feline tumors (1–3). Some of this rarity is likely attributed to the fact that many female companion animals are ovariohysterectomized at a young age. The most commonly reported reproductive tract neoplasms include ovarian carcinomas, granulosa-theca cell tumors (GTCT), and uterine tumors including benign leiomyomas and fibromas. More uncommon are malignant neoplasms such as leiomyosarcomas, fibrosarcomas, and adenocarcinomas (1). There is no reported data on the number of cases of canine or feline oviductal neoplasms to date. Malignant oviductal leiomyosarcoma is an exceedingly rare diagnosis in canines. Case reports of oviductal leiomyosarcoma have only been reported in lines of egg-producing Japanese quail, a single Blue-Fronted Amazon Parrot, and women at the time of this publication (4–7). Avian species are oviparous and have a much more robust oviduct that is responsible for egg production constituting the majority of their reproductive tract. The utility of these species in comparison to canines is limited. This report aims to describe a clinical case of oviductal leiomyosarcoma in a bitch.

## Case presentation

An 11-year-old, intact female Pomeranian was presented to the small animal emergency service for lethargy of 18 h duration with symptoms of anorexia, reluctance to move, and a single episode of inappropriate urination in the house. The patient was reported to be drinking and defecating less than normal. She had a history of intermittent licking of her perineal region and straining to defecate which was symptomatically treated with the addition of canned pumpkin to her diet. She was not on any medications and did not consistently receive flea, tick, or heartworm prevention. On the initial physical exam, the dog appeared quiet, alert, and responsive. On initial presentation, she was febrile (40°C) with normal heart rate, respiratory rate, and effort. A 1 mm inactive circular scar was noted on the right corneal surface, a grade II out of VI left systolic heart murmur was appreciated, the haircoat was dry and dull with hypotrichosis over the ventrum, and the patient had mild diffuse muscle atrophy. Digital and speculum vaginal exams were not performed on the initial exam.

Blood was submitted for a complete blood count (CBC) and serum chemistry panel. An abdominal-focused assessment with sonography (AFAST) was performed and a 3×3 centimeter mass of mixed echogenicity in the proximity of the left ovary was identified. A urine sample was collected via cystocentesis and submitted for urinalysis. On complete blood count, the red blood cell count was mildly decreased (5.31 × 10^6/uL; reference range 5.65–8.87 × 10^6/uL), hemoglobin was mildly decreased (12.8 g/dL; reference range 13.1–20.5 g/dL), and hematocrit was mildly decreased (37.1%; reference range 37.3%–61.7%). This was consistent with a mild normocytic normochromic nonregenerative anemia and likely reflected anemia of chronic inflammatory disease. Total white blood cell count was mildly increased (18.56 × 10^3/uL; reference range 5.05–16.76 × 10^3/uL) with increased segmented neutrophils (15.59 × 10^3/uL;), and increased platelets (531 × 10^3/uL; reference interval 148–484 × 10^3/uL), all suggestive of an inflammatory response. On serum chemistry, there was mild hypoglycemia (78 mg/dL; reference interval 88–121 mg/dL), increased blood urea nitrogen (35 mg/dL; reference interval 9–30 mg/dL), hypocalcemia (8.9 mg/dL; reference interval 9.4–10.7 mg/dL), increased alkaline phosphatase (88 U/L; reference interval 8–70 U/L), and increased anion gap (18.7 mEq/L, reference interval 12.3–18.5 mEq/L). These changes were likely related to anorexia, mild dehydration, urinary disease, azotemia (pre-renal and renal component), and mild age-related degenerative changes to the liver. On urinalysis, from cystocentesis collected sample, the urine specific gravity was 1.012, pH was 7.0, and there was 2+ protein, 2+ blood, 1–9 RBC/hpf, 3–32 WBC/hpf, 1–9 epithelial cells/hpf, and large numbers of bacteria/hpf. These findings were consistent with bacterial cystitis. A sterile urine sample was submitted for culture and grew >100,000 CFUs/mL of *Escherichia coli* susceptible to multiple first-line antimicrobials. Treatment with Clavamox (amoxicillin and clavulanate potassium) liquid suspension at appropriate dosing was initiated to treat the urinary tract infection. It was suspected that the larger mass visualized on ultrasound was suggestive of a reproductive tumor or cystic structure, but abdominal exploratory surgery was needed to confirm. No other pre-operative imaging was performed due to the patient’s clinical status, concern for the status of the uterus and possible pyometra, and lack of a definitive diagnosis of neoplasia. The patient was scheduled for ovariohysterectomy and abdominal exploratory surgery two days later with the small animal theriogenology service.

**Figure 1:**
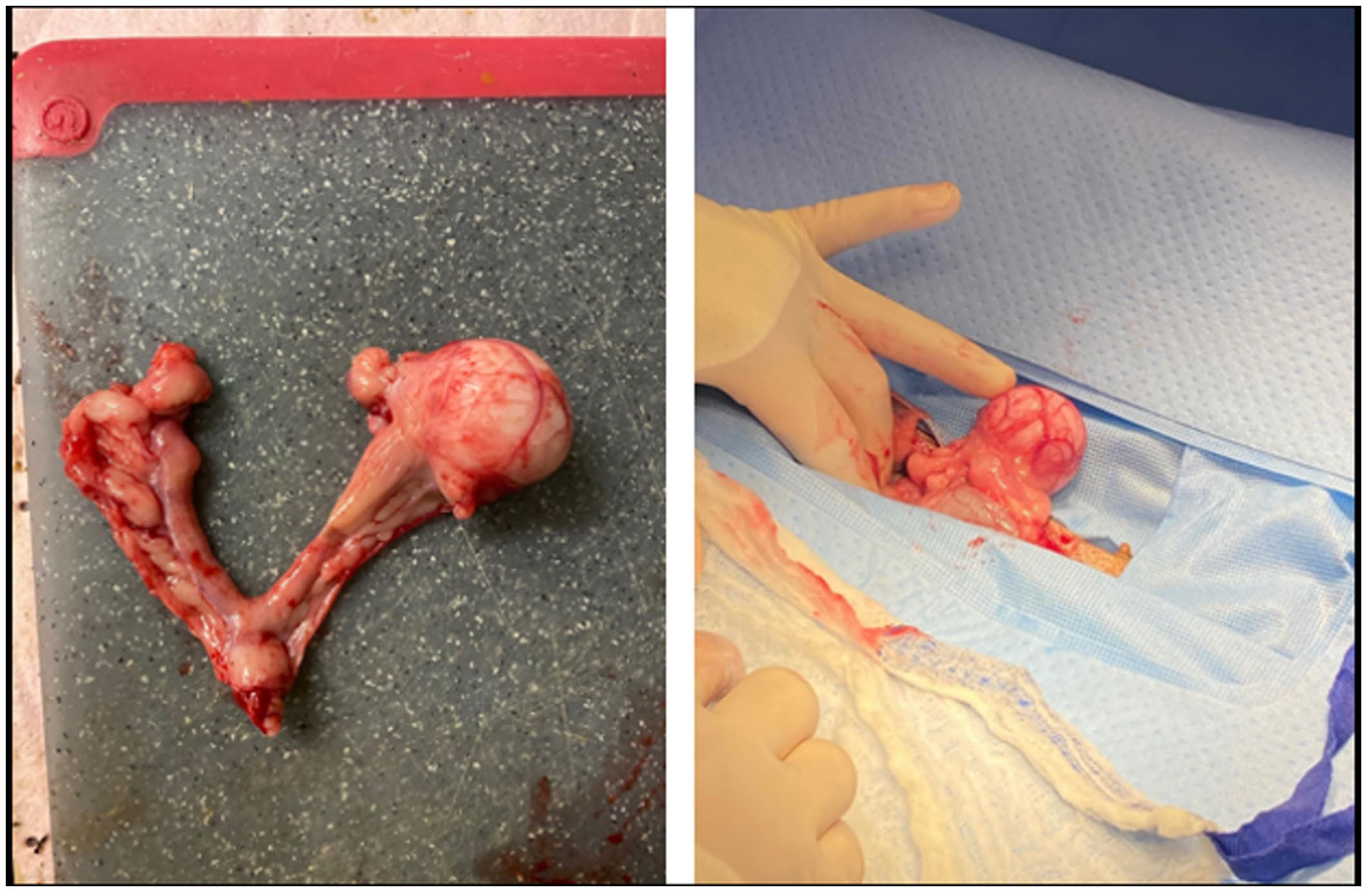
Excised uterus and ovaries from the patient. The left and right ovaries appeared grossly normal and are identified by the black arrows. The 3 cm left oviductal mass is identified by the black star. The 5 mm mass in the right uterine horn is identified by the black triangle. The uterine body and cervix appeared grossly normal and are identified by the black circle.

The patient returned for ovariohysterectomy and abdominal exploratory surgery two days later. The patient was reportedly doing better at home since starting the antibiotics. She was bright, alert, and responsive and her vital parameters were within normal limits on presentation for surgery. In addition to the previous findings, it was additionally noted that the patient had clitoral hyperplasia as well as a 0.5 cm dermal nodule on the right side of the labia. The nodule did not extend into the vulva. The patient was premedicated and anesthetized routinely with abdominal laparotomy performed via ventral midline incision. The left ovary was identified and visualized. An approximately 3 cm round mass was visualized near the cranial aspect of the left uterine horn (Figure 1). The ovary itself appeared grossly normal with multiple follicles present. The right ovary was identified and appeared grossly normal. In addition, an approximately 5 mm mass was identified near the cranial region of the right uterine horn. The uterine body was identified, visualized, and gently palpated for any abnormalities and none were appreciated. The remainder These findings were consistent with multiple reproductive tract masses and the remainder of the ovariohysterectomy was performed routinely with the masses being completely excised within the reproductive tract. A brief abdominal exploratory was performed and no other lesions or abnormalities were appreciated. There were no surgical complications and the dog recovered from anesthesia uneventfully.

**Figure 2:**
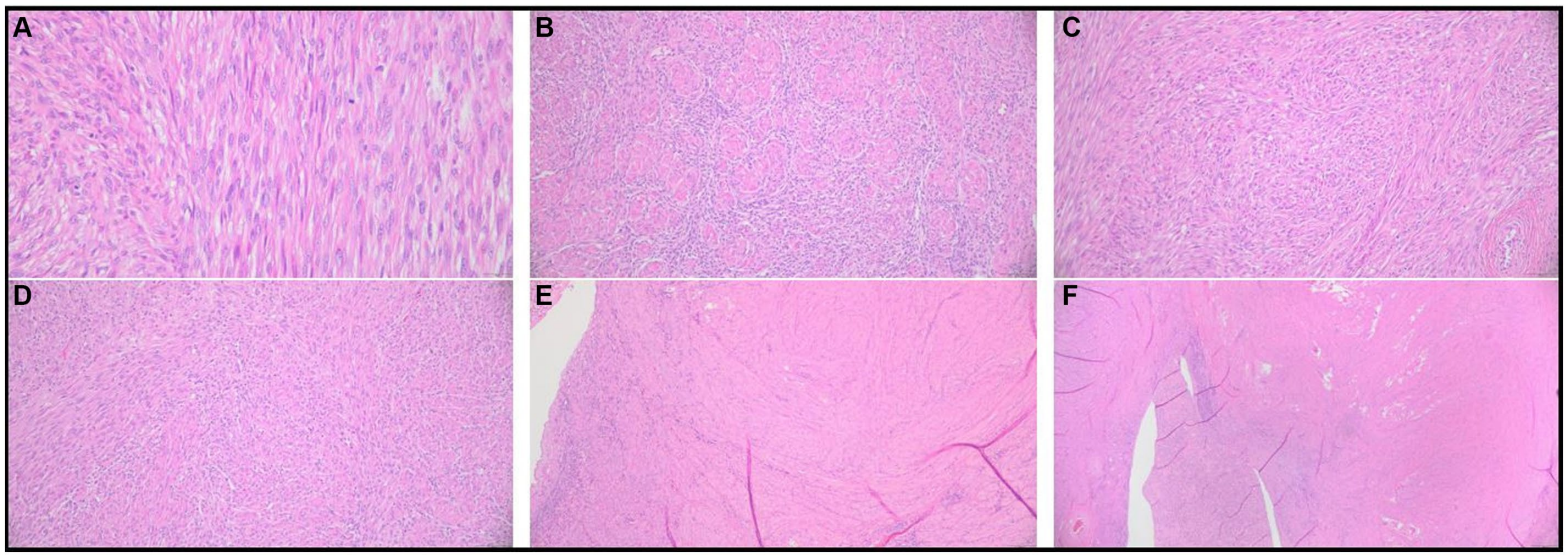
Histopathological results of excisional biopsy highlighting grade 1 uterine and oviductal leiomyosarcoma. Photomicrographs of hematoxylin and eosin-stained preserved tissue samples. **(A)** Spindle-shaped cells with indistinct cellular borders, eosinophilic cytoplasm, and eosinophilic cellular matrix. **(B–E)** Abnormal spindle-shaped cells are organized in sheets and bundles. **(F)** Abnormal spindle cell population infiltrating the oviductal and uterine smooth muscle tissue.

**Figure 3:**
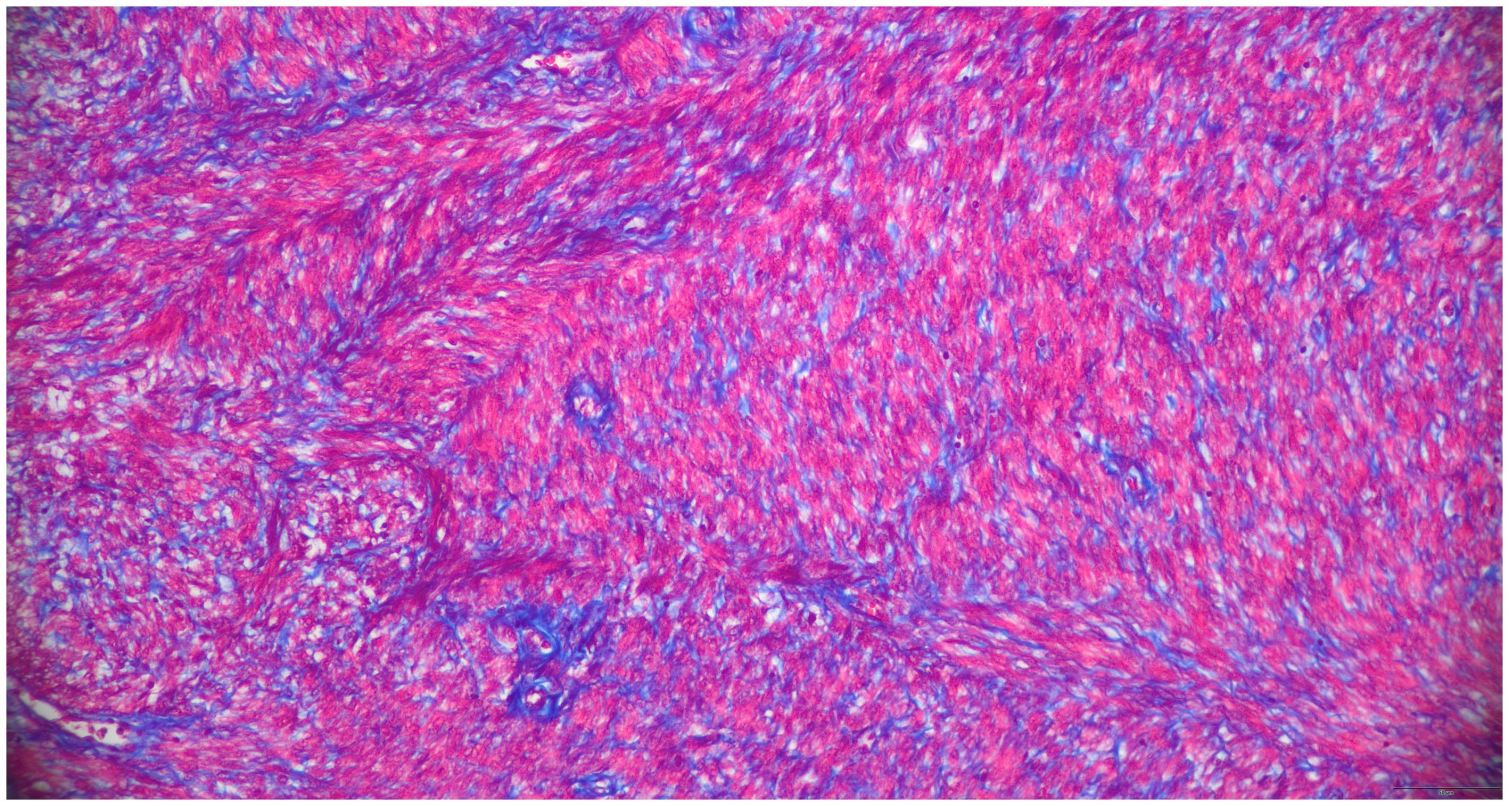
Histopathologic results of excisional biopsy highlighting tissue distinction. 200x, Masson’s Trichrome. The smooth muscle sarcoplasm is red, perivascular and stromal mesenchymal cells are blue.

The 3 cm and 5 cm grossly observable masses were adjacent to the ovaries and blended with smooth muscle in the area of the presumed oviducts and uterus (Figure 2). These masses were comprised of spindle-shaped cells in sheets and bundles with eosinophilic cytoplasm, indistinct cellular borders, and abundant eosinophilic matrix. The nuclei were oval to elongate, uniformly sized, and had blunted edges. The mitotic count was 2 in 2.37 mm^2^. Histochemical staining using Masson’s Trichrome demonstrated sheets of smooth muscle cells surrounded by stromal mesenchymal cells (Figure 3). These characteristics, are consistent with grade 1 oviductal and uterine leiomyosarcoma, a malignant neoplasm of the smooth muscle tissue in the oviduct and uterus. One of the ovaries also contained a small, cellular mass that was not grossly observed, which was compressing developing follicles to the periphery. This ovarian mass was incidentally found on histopathology. The ovarian mass was formed by polygonal cells with eosinophilic cytoplasm and indistinct cellular borders arranged in papillary structures. This mass was consistent with an ovarian papillary adenoma, a benign neoplasm of ovarian glandular tissue. The uterus was affected diffusely by cystic endometrial hyperplasia with areas of invasion into the tunica muscularis characterized by cellular debris and neutrophils infiltrating the lumen of the cysts.

On follow-up telephone communications three days postoperatively, the dog was reported to be in good condition and recovering well with no concerns. Further staging, including diagnostic imaging, one month from the procedure was offered in the event of local metastasis, but was not pursued by the dog’s owners. At this time of this report, 8 months post-surgery, no follow-up appointments have been performed with the VMCVM. The patient was lost to follow up for telecommunication and any additional information.

## Discussion

Leiomyosarcoma is a malignant neoplasm of smooth muscle tissue. This neoplasm is most commonly reported to affect the smooth muscle tissue of the gastrointestinal tract, urogenital tracts, liver, and spleen in veterinary patients (8). As noted previously, reproductive tract neoplasia is uncommon in canine and feline veterinary patients. In a study characterizing urogenital tumors diagnosed at a Polish veterinary teaching hospital, 38% of feline urogenital tumors diagnosed were localized to the uterus and 2.5% of diagnosed canine urogenital tumors were localized to the uterus (9). In the cases recorded in the veterinary literature, mesenchymal neoplasms, leiomyomas, and leiomyosarcomas make up 95%–100% of these relatively rare canine uterine neoplasms (1, 9). Benign leiomyomas, known as uterine fibroids in human medicine, make up the majority of uterine mesenchymal tumors in canines and felines. Less common leiomyosarcomas have been reported to account for 10% of uterine tumors in dogs (10). The majority of cases of reproductive neoplasia occur in intact females, however, rare cases of reproductive neoplasia have been reported to arise in ovariohysterectomized patients (9, 11–13). No specific breed of dog or cat has been described to be at increased risk of reproductive leiomyosarcoma.

Common clinical signs of patients with leiomyosarcoma depend on the site of the neoplasm.

Clinical signs of uterine leiomyosarcoma, when present, are consistent with those of other reproductive tract neoplasms. These can include abdominal distension, compression of surrounding structures (resulting in stranguria or constipation), abnormal estrus cycles, vaginal discharge, pyometra, polyuria, polydipsia, vomiting, and weight loss (1, 2, 11–13). These clinical signs are often appreciated when the tumor has grown to a considerable size (1). Often, however, these tumors are diagnosed incidentally on ovariohysterectomy of affected patients without overt clinical signs (1, 9). In our case, the patient presented with consistent clinical signs of tenesmus. The tenesmus could be a result of mechanical compression of the masses on the colon. However, it could also be secondary to diet, stranguria secondary to bacterial cystitis, or dehydration, all of which this patient had as clinical signs during the initial presentation.

Gastrointestinal leiomyosarcomas such as leiomyosarcoma of the liver, stomach, duodenum, or jejunum can cause hypoglycemia, which is suggested to be related to glucose consumption by the tumor, liver damage related to neoplasia, secretion of an insulin-like compound by the tumor, or concurrent peritonitis and sepsis due to impaired intestinal wall integrity and perforation. To the best of our knowledge this phenomenon has not been reported in leiomyosarcoma of the reproductive tract, but our patient did present with hypoglycemia (14–16). Alternatively, this hypoglycemia could be secondary to another disease process that was not identified in the case work up. Bloodwork was not collected to recheck hypoglycemia post-operatively and the patient was lost to follow up, so it is difficult to further evaluate the cause of this patient’s hypoglycemia and the response to surgical intervention.

Reproductive tract tumors, when suspected before surgery, are often visualized on abdominal imaging (1). Abdominal radiographs and abdominal ultrasound are the most common first-line imaging modalities used, but these tumors may also be diagnosed on advanced imaging such as a CT scan. Imaging is helpful for the staging of neoplasia and thoracic radiographs should be obtained before the removal of a suspected tumor as metastatic disease will alter the patient’s prognosis. The only way to definitively diagnose a reproductive neoplasm is to sample the tumor with a fine needle aspirate, obtain an incisional biopsy, or perform ovariohysterectomy and submit tissue for histopathological analysis (1).

The treatment of choice for most animals with suspected reproductive neoplasia is ovariohysterectomy. Due to the locally invasive behavior of leiomyosarcoma, the neoplasm of interest in this publication, surgical resection of the affected reproductive tract via ovariohysterectomy is often successful in treating this condition if metastasis has not occurred (1). Leiomyosarcomas overall are reported to have a 50% incidence of metastasis and in one study of cats with uterine leiomyosarcoma, metastases were present in two out of three diagnosed cases (2, 17). In one reported case of myxoid uterine leiomyosarcoma, a subtype of uterine leiomyosarcoma, documented in a cat widespread sarcomatosis occurred 30 days post-ovariohysterectomy. This led to the euthanasia of the cat to prevent further suffering (3). This is suggested to be a more aggressive subtype of leiomyosarcoma similar to the behavior of the same neoplasm, myxoid uterine leiomyosarcoma, that is seen in human patients (3).

There is little known information on the efficacy of chemotherapy and radiation therapy in the treatment of this condition as the post-operative behavior of uterine leiomyosarcoma is not well defined. These treatment methods could be considered an option if full surgical resection is not possible (1). In human patients with high-grade uterine leiomyosarcoma recurrence after surgical resection is between 50%–70% (18). Due to this rate of recurrence adjuvant therapy such as chemotherapeutics, radiation therapy, and hormonal blockade therapy have been used in human patients with uterine leiomyosarcoma. None of these treatments are proven to significantly reduce the risk of relapse or improve survival times in human patients (18). Some of the aforementioned adjuvant therapies, however, were found to be beneficial only in advanced stages of human uterine leiomyosarcoma (18).

In this case, a benign ovarian papillary adenoma was incidentally resected with the affected ovary and diagnosed via histopathology. Ovarian tumors are uncommon in dogs as previously discussed. Epithelial tumors are the most common subtype in dogs and of these epithelial tumors including adenocarcinomas, rete adenomas, papillary adenomas, and cystadenomas, malignant tumors are the most common (1). In cats, however, sex cord stromal tumors are reported to be the most prevalent ovarian neoplasm (1). Most ovarian epithelial tumors are bilateral, but in our case the tumor was unilateral (19). Clinical signs associated with ovarian tumors are mostly related to the space occupied by the lesion leading to weight loss, lethargy, vomiting, ascites, and abdominal distension, and the production of sex hormones by ovarian tumors is reported (1). In this case, the papillary adenoma was small and unlikely to cause discomfort. It is reported that epithelial ovarian tumors are unlikely to produce sex hormones as compared to other types of ovarian neoplasia (20, 21). The patient in this report did have clitoral hypertrophy and cystic endometrial hyperplasia, so it is possible that this could be related to hormone production by the benign ovarian lesion and/or due to age-related changes with sustained hormonal influence. There is no evidence found at the time of publication that smooth muscle neoplasms of the uterus and oviduct have been known to produce sex hormones.

The patient also had evidence of cystic endometrial hyperplasia (CEH) on histopathological analysis of uterine tissue. This finding is a common hormonally responsive change to the uterine wall in older bitches. In a study of 240 bitches ranging in age from 1–7 years in Europe, 18.3% of bitches were diagnosed with CEH on ultrasound. This percentage increased with age as 6.8% of 2-year-old bitches had CEH while 60% of 6-year-old bitches had CEH (20). Due to the high incidence of CEH in intact dogs and the relatively low incidence of uterine and oviductal neoplasms in canines, it is unlikely that the CEH lesions in this patient were related to the diagnosed leiomyosarcoma in this case.

## Concluding remarks

This report documents the diagnosis and treatment of a dog diagnosed with oviductal and uterine leiomyosarcoma. This case highlights the rare, but successful diagnosis and treatment of these reproductive neoplasms.

## Data availability statement

The original contributions presented in the study are included in the article/Supplementary material, further inquiries can be directed to the corresponding author.

## Ethics statement

This study was carried out in accordance with the principles of the Department of Small Animal Clinical Sciences at the Virginia-Maryland College of Veterinary Medicine and the patient was provided best practice veterinary care.

## Author contributions

JK and NS contributed to writing the manuscript and literature review. TL assessed the gross and histopathologic findings. KW and NS performed the surgery and managed the clinical case. JC contributed to the critical revision of the manuscript. All authors contributed to the article and approved the submitted version.
